# Geometric optimization of an ultralow-dose high-resolution pediatric PET scanner based on monolithic scintillators with dSiPM readout

**DOI:** 10.1186/2197-7364-2-S1-A23

**Published:** 2015-05-18

**Authors:** Ekaterina Mikhaylova, Valerio Tabacchini, Giacomo Borghi, Pieter Mollet, Ester D‘Hoe, Dennis R Schaart, Stefaan Vandenberghe

**Affiliations:** MEDISIP, Department of Electronics and Information Systems, Ghent University, Belgium; iMinds Medical IT, Ghent, Belgium; Radiation Science & Technology, Technical University Delft, Netherlands

A potential design for a high-resolution, ultralow-dose multimodal PET insert for pediatric medicine is presented. Several new technologies are combined to develop a unique, mobile, CT- and MRI-compatible PET system. The system is intended for diagnosis, staging, monitoring, and follow-up in the treatment of cancer as well as cardiac and neurological diseases, inflammation, and hyperinsulinism in children of up to about 12 years of age. The design is based on a recently developed monolithic scintillator detector, consisting of a 32 x 32 x 22 mm^3^ LYSO:Ce crystal with dualsided readout (DSR) using digital silicon photomultiplier (dSiPM) arrays. The aim is to achieve an isotropic spatial resolution of < 2 mm full width at half maximum (FWHM) in the entire field-of-view (FOV), as well as < 150 ps FWHM time-of-flight (TOF) resolution. The central goal of this work is the simulation, geometric design optimization, and preliminary performance evaluation of the pediatric system. The scanner geometry is simulated by means of the GEANT4 Application for Tomographic Emission (GATE) software, using the measured spatial-, energy- and timing-response of the DSR monolithic scintillator detectors as input. The performance of pediatric PET inserts with different axial lengths (between 6.8 cm and 102 cm) is assessed following the NEMA NU2-2012 protocol. Preliminary results show that the system can achieve a spatial resolution as good as ~2.0 mm FWHM in each direction, a scatter fraction (SF) of ~32%, and a line source sensitivity as high as ~188 cps/kBq for 1 m long scanner. Further work includes the simulation of different potential geometries of the pediatric PET and the comparison of their performance. Additionally, a more complete and accurate model of the detector response based on experimental data will be implemented for better spatial resolution.

